# *Listeria monocytogenes* ST37 Distribution in the Moscow Region and Properties of Clinical and Foodborne Isolates

**DOI:** 10.3390/life13112167

**Published:** 2023-11-05

**Authors:** Olga L. Voronina, Marina S. Kunda, Natalia N. Ryzhova, Ekaterina I. Aksenova, Margarita A. Kustova, Tatiana I. Karpova, Alina R. Melkumyan, Elena A. Klimova, Olga A. Gruzdeva, Igor S. Tartakovsky

**Affiliations:** 1 N.F. Gamaleya National Research Center for Epidemiology and Microbiology, Ministry of Health of the Russian Federation, Gamaleya Str., 18, 123098 Moscow, Russia; markunda99@gmail.com (M.S.K.); rynatalia@yandex.ru (N.N.R.); aksenova16@yandex.ru (E.I.A.); kystowaa@yandex.ru (M.A.K.); dragovtceva@yandex.ru (T.I.K.); itartak@list.ru (I.S.T.); 2 F.I. Inosemtsev City Clinical Hospital, Fortunatovskaya Str., 1, 105187 Moscow, Russia; alinamelkumyan@yandex.ru; 3 Department of Infectious Diseases and Epidemiology, A.I. Yevdokimov Moscow State University of Medicine and Dentistry, Ministry of Health of the Russian Federation, Delegatskaya Str., 20, Building 1, 127473 Moscow, Russia; elena_klimova_@mail.ru; 4 Federal State Budgetary Educational Institution of Further Professional Education Russian Medical Academy of Continuous Professional Education, Ministry of Health of the Russian Federation, Barrikadnaya Str., 2/1, Building 1, 125993 Moscow, Russia; gruzdeva_oa@mail.ru

**Keywords:** *Listeria monocytogenes*, foodborne pathogen, invasive listeriosis, cgMLST, whole genome sequencing, prophages, virulence factors, COVID-19

## Abstract

*Listerias* of the phylogenetic lineage II (PLII) are common in the European environment and are hypovirulent. Despite this, they caused more than a third of the sporadic cases of listeriosis and multi-country foodborne outbreaks. *L. monocytogenes* ST37 is one of them. During the COVID-19 pandemic, ST37 appeared in clinical cases and ranked second in occurrence among food isolates in the Moscow region. The aim of this study was to describe the genomic features of ST37 isolates from different sources. All clinical cases of ST37 were in the cohort of male patients (age, 48–81 years) with meningitis–septicemia manifestation and COVID-19 or Influenza in the anamnesis. The core genomes of the fish isolates were closely related. The clinical and meat isolates revealed a large diversity. Prophages (2–4/genome) were the source of the unique genes. Two clinical isolates displayed pseudolysogeny, and excided prophages were A006-like. In the absence of plasmids, the assortment of virulence factors and resistance determinants in the chromosome corresponded to the hypovirulent characteristics. However, all clinical isolates caused severe disease, with deaths in four cases. Thus, these studies allow us to speculate that a previous viral infection increases human susceptibility to listeriosis.

## 1. Introduction

*Listeria monocytogenes* is ubiquitously distributed in the environment as a saprophyte. The population of *L. monocytogenes* consists of four divergent lineages (I–IV) [1]. *L. monocytogenes* of the phylogenetic lineages (PL) I and II are the actual foodborne pathogens and cause the majority of human illness. *L. monocytogenes* of the PLI, which was introduced into Europe (for example, clonal complex 1 (CC1) around 1868) [2], has hypervirulent clones CC1, CC2, CC4, and CC6 with high clinical frequency [3]. At the same time, the clones of *L. monocytogenes* of the PLII are common for the European environment, are hypovirulent, and infect mostly highly immunocompromised individuals [3]. In the largest European collection of *L. monocytogenes* collected in France between January 2005 and October 2013, isolates of the PLI accounted for 66%, and isolates of the PLII accounted for 34% of the total clinical isolates [4]. The isolates of ST37 accounted for 5% of the clinical isolates in the PLII [4]. It was in seventh place after CC8-16, CC9, CC121, CC7, CC155, and CC101-90 [4]. The first registered clinical isolates of ST37 were mentioned by Ragon et al. in two cases of the maternal–neonatal (MN) infection in 1991 [5]. In the LiSEQ (*Listeria* SEQuencing) study of the 1143 *L. monocytogenes* isolates collected during the EU-wide baseline survey in 2010 and 2011, the isolates of ST37 accounted for 2% of the clinical, sporadic isolates [6].

There are some sources of *L. monocytogenes* ST37. First, it is the environment. So Linke et al. demonstrated that *L. monocytogenes* ST37, along with ST517, ST91, and ST101, could be repeatedly isolated from soil samples in two regions near the Schwarza and Danube rivers over several months [7]. *L. monocytogenes* fecal carriage occurs in clinically healthy ruminants (domestic and wild), swine, ground game, and birds [8,9,10]. These animals are potentially important reservoirs for *L. monocytogenes* and shed bacteria with feces in the environment. ST37 was one of the most frequent STs found in different sample types of hunted game and game meat in Finland between 2012 and 2020 [10]. *L. monocytogenes* ST37 was the third most common on Finnish dairy farms from 2013 to 2016 [11]. On 27 meat and dairy cattle farms in Latvia, CC37 was one of the predominant *L. monocytogenes* clones from March 2019 to August 2020 [12].

In Slovakia, *L. monocytogenes* ST37 was isolated from animals with clinical manifestations of listeriosis from FPE (food processing environment), meat, and dairy products, with CC37 being one of the most prevalent in the milk sector [13]. On the food production plants, *L. monocytogenes* ST37 was isolated in Germany (2008–2016) [14], in Austria (the meat processing facility, 2013–2018) [15], and in Spain (the new meat processing facility, 2017) [16]. In Russia, *L. monocytogenes* ST37 was earlier isolated from meat and dairy products [17], but clinical isolates of this genotype have never been reported in invasive listeriosis.

The crucial change in the spectrum of the *L. monocytogenes* ST causing invasive listeriosis was indicated during the COVID-19 pandemic [18]. Now patients who have had COVID-19 have become sensitive to *L. monocytogenes* ST8, ST21, ST37, ST391, and ST425. The range of products from which *L. monocytogenes* ST37 was isolated has been expanded [18].

Thus, *L. monocytogenes* ST37 required special attention. The present study is devoted to the analysis of isolates of this genotype from different sources.

## 2. Materials and Methods

### 2.1. Materials

Isolates of *L. monocytogenes* ST37 were collected during the multicenter monitoring of *L. monocytogenes* in the Moscow region between November 2018 and February 2023 and were submitted to the Bacterial Isolate Genome Sequence Database for *L. monocytogenes* (BIGSdb-Lm) (https://bigsdb.pasteur.fr/listeria/ (accessed on 5 September 2023)), ID: 76308; 78656; 82484; 98284; 98285; 98295; 102091 (clinical isolates); 42997; 42998; 49371; 49372; 76388; 82493; 98277; 98278; and 98280 (foodborne isolates).

### 2.2. Methods

#### 2.2.1. DNA Isolation

For genotyping, listeria isolates were grown on BHI agar overnight at 37 °C. One loopful of cells was suspended in 20 µL of the lysis buffer (0.25% SDS and 0.05 M NaOH), warmed up for 15 min at 95 °C, and 180 µL of the bidistilled water was added. The resulting lysate was stored at a temperature of 4 °C. For the whole genome sequencing, listeria isolates grown on BHI agar overnight at 37 °C were suspended in the BHI broth and grown overnight at the same temperature. The cells were centrifuged, and the washed pellet was used for DNA isolation according to the Monarch Kit instructions (New England Biolabs, Ipswich, MA, USA).

#### 2.2.2. MultiLocus Sequence Typing

MultiLocus Sequence Typing (MLST) and Multi-Virulent-Locus Sequence Typing (MvLST) were performed as described earlier [18]. The MvLST scheme included four internalin genes (inlABCE). The combination of four detected alleles (in order, inlABCE) received an internalin profile (IP) number.

#### 2.2.3. Whole Genome Sequencing (WGS)

The genomes of the strains with ST37 were sequenced on the Illumina platform. The Nextera DNA Flex Library Prep (Illumina, San Diego, CA, USA) protocol and the NadPrep EZ DNA Library Preparation protocol (Nanodigmbio (Nanjing) Biotechnology Co. Ltd., Nanjing, China) were used for the library’s preparation. Sequencing was performed on MiSeq and NextSeq 500/550 (Illumina, San Diego, CA, USA).

#### 2.2.4. Data Analysis

CLC Genomic Workbench v.21.0.1 (QIAGEN, Germantown, MD, USA) and SPAdes v.3.13.0 (St. Petersburg genome assembler, Russia, URL: http://cab.spbu.ru/software/spades/ (accessed on 5 September 2023)) were used for genome assembling. CGView Server (http://stothard.afns.ualberta.ca/cgview_server/ (accessed on 5 September 2023)) was applied for the visualization of assembling results and for the genome comparison [19]. The software Rapid Annotations Subsystems Technology (RAST) and SEED [20] and the NCBI Prokaryotic Genome Annotation Pipeline (PGAP) [21] were used for genome annotation. Prophage sequences were revealed with the help of PHASTER (PHAge Search Tool Enhanced Release, https://phaster.ca/ (accessed on 5 September 2023)) [22].

WGS data are available in GenBank: Bio Project PRJNA605697, accession numbers: CP127186–CP127191, CP127193, CP127240, CP127241, CP127377, and CP129425–CP129430.

The core genome MLST scheme of 1748 loci (cgMLST) was used for *L. monocytogenes* genome characterization [23] on the basis of an open bioinformatics platform (https://bigsdb.pasteur.fr/listeria/ (accessed on 5 September 2023)). Alleles that were not characterized with the computational tool due to their differences from the registered sequences were searched by comparing genomic data with alleles of the reference EGD-e strain using BLAST (2.10.0+) [24]. The identified sequences of the corresponding alleles of the isolates of the same ST were compared by the number of differences with the closest match. New alleles were considered identical if there were the same substitutions.

The phylogenetic tree on the base of the concatenated core genes was constructed using the neighbor-joining method with the help of the Whole Genome Alignment Plugin in CLC Genomic Workbench v.21.0.1 (QIAGEN, Germantown, MD, USA) and was represented in the MEGA7 program [25].

Pan genomic studies of the ST37 isolates were made with BPGA: Bacterial Pan Genome Analysis Pipeline (BPGA-Version-1.3) and included determining core (conserved), accessory (dispensable), and unique (strain-specific) genes with the KEGG (Kyoto Encyclopedia of Genes and Genomes) and COG (*Clusters of Orthologous Genes*) mapping [26]. The analysis was performed with the following parameters: clustering was made with USEARCH, amino acid identity cutoff = 50%, and the type of phylogeny tree was neighbor joining.

Prophages regions were identified using PHASTER (an extended version of the PHAge search tool, https://phaster.ca/ (accessed on 5 September 2023)) [27].

CRISPR (clustered regularly interspaced short palindromic repeats) arrays and their associated (Cas) proteins were identified with the help of the CRISPRCasFinder (https://crisprcas.i2bc.paris-saclay.fr/ (accessed on 5 September 2023)) [28].

The virulence factor database (VFDB) (http://www.mgc.ac.cn/VFs/ (accessed on 5 September 2023)), an extensive collection of VFs from the important bacterial pathogens [29], as well as the BIGSdb-Lm database (https://bigsdb.pasteur.fr/listeria/ (accessed on 5 September 2023)) were used for the VFs analysis of *L. monocytogenes* ST37.

The resistome of the *L. monocytogenes* ST37 isolates was analyzed using the Antibiotic Resistance BIGSdb-Lm database (https://bigsdb.pasteur.fr/listeria/ (accessed on 5 September 2023) and the data from the Comprehensive Antibiotic Resistance Database (https://card.mcmaster.ca/ (accessed on 5 September 2023)) [30].

We used all bioinformation resources with default parameters.

#### 2.2.5. Ethical Approval

The study was carried out with the informed consent of the patients. The research protocol was approved by the Biomedical Ethics Committee of the N.F. Gamaleya National Research Center for Epidemiology and Microbiology (protocol No. 14, 4 July 2018).

## 3. Results

**Figure 1:**
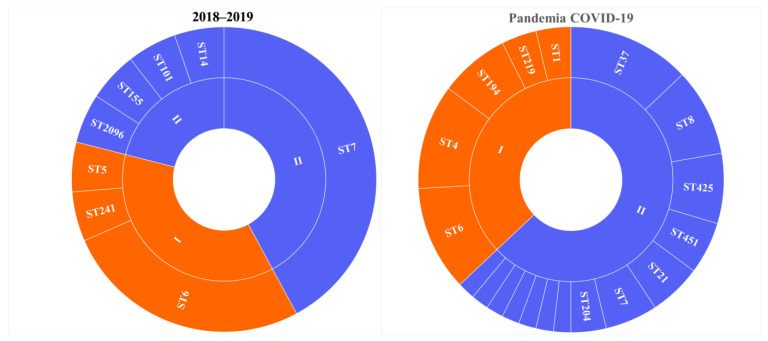
Genetic diversity of *L. monocytogenes* isolated from patients with invasive listeriosis (**A**) before and (**B**) during the COVID-19 pandemic. I - phylogenetic lineage I, II - phylogenetic lineage II.

The COVID-19 pandemic revealed a large diversity of *L. monocytogenes* of the PLII in clinical cases (Figure 1) [18]. *L. monocytogenes* ST37 that appeared during the pandemic took first place and shared 13% among all clinical isolates (Figure 1) and 21% among isolates of the PLII.

### 3.1. Prevalence of L. monocytogenes ST37

**Figure 2:**
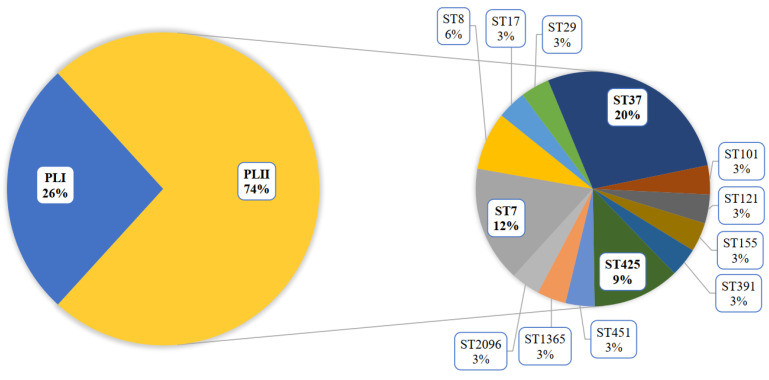
Genotype diversity of *L. monocytogenes* isolated from male patients with invasive listeriosis.

The greatest contribution to the diversity of the PLII genotypes was made by patients with meningitis and septicemia. A total of 72% of patients in this cohort were male. All cases of *L. monocytogenes* ST37 were noted in this group. The male patients were mostly infected with *L. monocytogenes* of the PLII (Figure 2), and ST37 shared 20% in this group.

The male patients infected by *L. monocytogenes* ST37 were aged 48–81 years (mean age—66 years) and had COVID-19 (the most cases) or Influenza A (one case) in their anamnesis. Only two patients survived.

**Figure 3:**
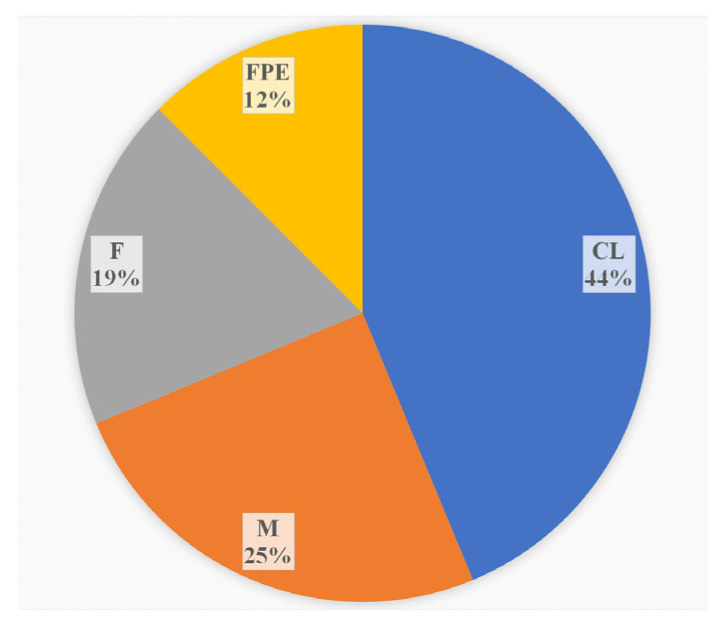
The sources of *L. monocytogenes* ST37 in the study. CL—clinical specimens, M—meat products, F—fish products, and FPE—restaurant cooking environments.

During the monitoring period, 2018–2023, in addition to clinical specimens, the sources of *L. monocytogenes* ST37 were fish and meat products and restaurant cooking environments (Figure 3). Overall, among food isolates, ST37 ranked second in occurrence after ST121.

### 3.2. Genomic Analysis of the L. monocytogenes ST37 Isolates

All isolates of *L. monocytogenes* ST37 were analyzed together via whole genome sequencing (WGS).

#### 3.2.1. Core Genome including 1748 Genes

**Figure 4:**
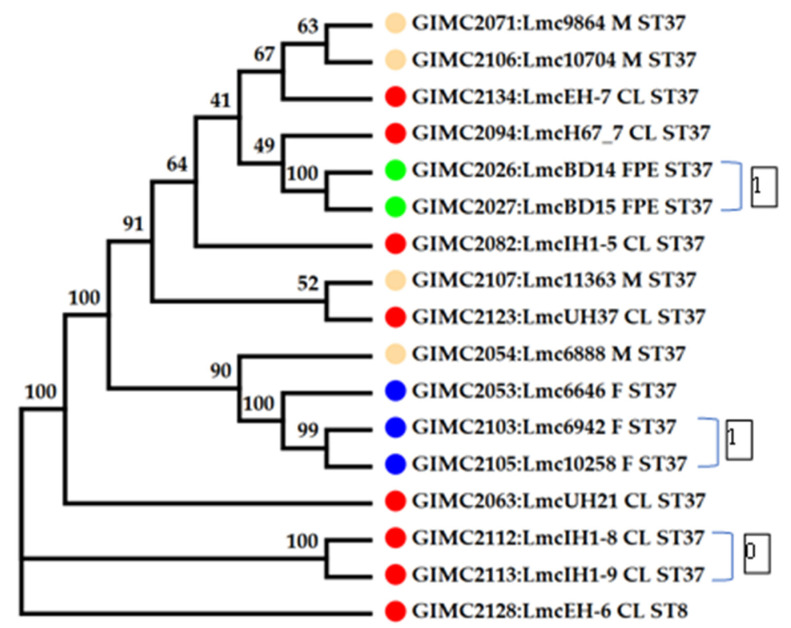
Phylogenetic tree at the base of the concatenated 1748 core genes. Colored circles for the source of isolates are indicated as follows: red—clinical, blue—fish, cream—meat, and green—the restaurant cooking environments. The number next to the bracket shows the loci of difference between a pair of core genomes. The numbers at the nodes indicate bootstrap values.

The cgMLST was performed on 16 genomes of *L. monocytogenes* ST37. The results of the comparison are shown in Figure 4 (Table S1). Only tree pairs of the genomes had null or one locus of difference. The pair of clinical isolates came from two samples of one patient: blood and brain membranes (postmortem). These isolates had the same core genomes.

The pair of FPE isolates had one locus of difference. The samples for their isolation were collected in one place and over one day. At last, the pair of fish isolates were from salmon fillets from one manufacturer, delivered two weeks apart. The third fish isolate differed from the isolates of the pair by 11–12 loci. Clinical isolates, all but two mentioned above, had 16–37 loci of differences. They differed from the pair of one patient’s isolates by 74–84 loci. The same differences were observed from this pair of clinical isolates from food and FPE. So, the pair of clinical isolates had an unknown source that was significantly different from the sources of other isolates.

#### 3.2.2. Pan Genomic Studies of the ST37 Isolates

**Figure 5:**
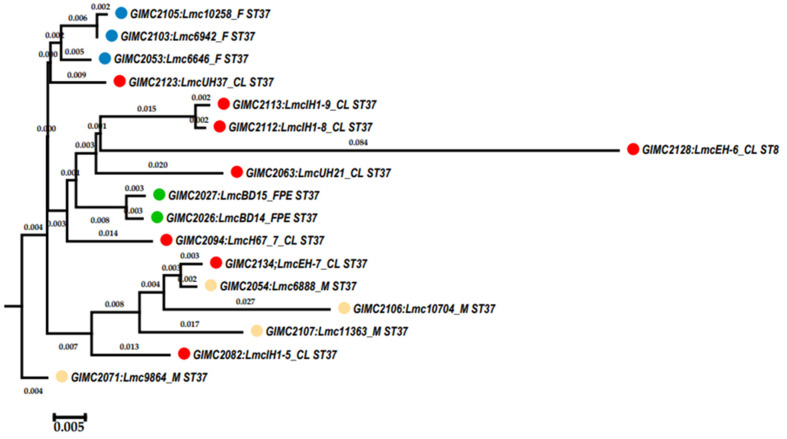
Phylogenetic tree generated with BPGA using 16 isolates of ST37 and 1 isolate of ST8 based on concatenated core genes. Colored circles for the source of isolates are indicated as follows: red—clinical, blue—fish, cream—meat, and green—the restaurant cooking environments. The numbers at the nodes indicate the length of the branches.

The pan genome and core genome sizes determined with the BPGA were 3189 and 2611, respectively. An increase in the core to 2611 genes changed the topology of some branches on the tree (Figure 5). In the phylogenetic group of the fish isolates, the branch of the clinical isolates appeared. Meat isolates, all but one, and two clinical isolates formed a separate phylogenetic group. 

**Figure 6:**
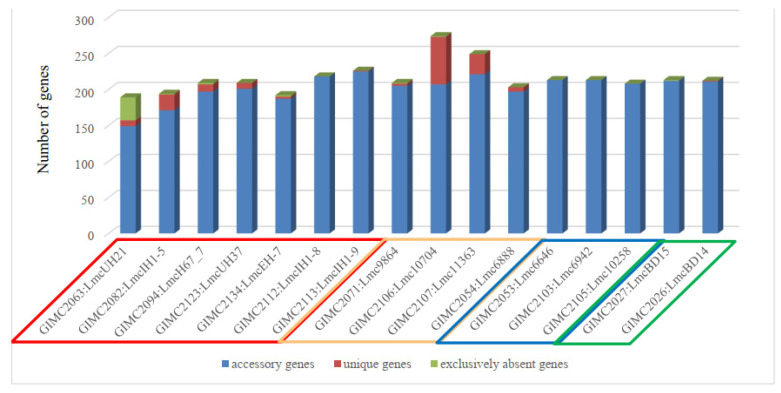
The genes outside the core genome. Colored frames for the source of isolates are indicated as follows: red—clinical, cream—meat, blue—fish, and green—the restaurant cooking environments.

The central phylogenetic group included the most different clinical isolates, the pair of the FPE isolates, and two other clinical isolates—one of which is LmcUH21 and has the smallest number of ORFs (open reading frames, 2840) and homologous genes (72) but also the largest number of exclusively absent genes (38) (Figure 6).

Meat isolates were the leaders in the number of unique genes, followed by some clinical isolates. Unique genes were absent in a group of fish and FPE isolates as well as in a pair of very different clinical isolates.

The distribution of the COG groups was different for core, accessory, and unique genes (Table 1). Since the core genes are responsible for the basic aspects of the biology of the species [26], it is not surprising that 40% of the core genes of *L. monocytogenes* ST37 belong to the “Metabolism” group. The genes responsible for “Cellular processes” and “Information storage” were the most abundant in the unique and accessory groups. Analysis of the location of unique genes in the genomes of *L. monocytogenes* ST37 showed that they are localized in the regions of prophages. Isolates Lmc10704 and Lmc11363 with the most prophages, — 4 (Table 2), had the highest number of unique genes (Figure 6), which confirms that prophages as mobile genetic elements are the sources of bacterial diversity [31].

#### 3.2.3. Prophages and Phages in the ST37 Isolates

There are some “hot spots” of genome evolution for *Listeria* spp. that are affected by the transduction processes of bacteriophages [32]. We found seven such regions in the genomes of *L. monocytogenes* ST37 (Table 2). Two of them were downstream loci of the regulators of transcription (lmo0112 and *comK*), and five regions were located adjacent to the tRNA genes. The lmo0112 questionable prophage, which is conserved across all lineages of *L. monocytogenes* [33], has been revealed in all genomes of *L. monocytogenes* ST37 (14.4 Kb) and harbors a complete *lma* operon (lmaDCBA) known as the monocin locus. The tRNA^Arg(tct)^ intact prophage was in all but one isolate (42.6–46.1 Kb). The next intact prophage, tRNA^Ser(cga)^, was 34.2Kb and has been found in all meats and one clinical isolate. The tRNA^Leu(gag)^ prophage inserted only in the meat isolate had the highest number of unique genes (questionable, 51.1 Kb). The tRNA^Arg(ccg)^ prophage was revealed in four isolates from different sources (intact 41.7 Kb or questionable 41.3 Kb). At last, the tRNA^Thr(ggt)^ prophage was found in all fish (intact 49.3 Kb) and in three clinical isolates (questionable, 53.1–57.9 Kb). The *comK* locus was disrupted via phage integration in two genomes (clinical and meat). Inserted prophages were intact (40.3 Kb) or questionable (49.9 Kb).

The tRNA^Thr(ggt)^ prophages of the one patient’s isolates (GIMC2112:LmcIH1-8 and GIMC2113:LmcIH1-9) had 59 ORF and were A006-like. WGS of these isolates demonstrated that genomes consisted of two replicons: chromosomes and bacteriophages of the class *Caudoviricetes*. The bacteriophages LP-LmcIH1-8 (OR234009) and LP-LmcIH1-9 (OR234010) had 66 and 65 ORF and were A006-like too. The tRNA^Thr(ggt)^ prophages and bacteriophages were organized from common functional modules [34]: (1) “late genes” coding for structural proteins, DNA packaging, and the lysis systems (holin and endolysin); (2) genes for lysogeny functions (integrase, repressor, and similar) and the prophage attachment and integration locus *attP*; and (3) “early genes” for the early state of phage reproduction encoding products for replication, recombination, and modification of the viral DNA. The tRNA^Thr(ggt)^ prophage and bacteriophage of one isolate had 19 identical ORF: 8 in the left arm of the bacteriophage genome, 6 in the right arm, and 5 ORF for phage proteins in the third module. The amino acid sequences of integrase, recombinase, holin, transcriptional regulators, and some phage proteins had the least similarity between prophage and phage. 

The WGS data indicated non-synchronous replication of the phage and the bacterial chromosome since the coverage of the phage replicon was 4–5 times greater than the coverage of the chromosome. Thus, we can assume there is pseudolysogeny, i.e., a type of phage–bacteria interaction in which the phage nucleic acid neither integrates into the host chromosome as a prophage (lysogeny) nor elicits a lytic response but resides within the cell in a non-active form [35,36]. Pseudolysogeny has been described for several tailed dsDNA phages [37], which include phages of the class *Caudoviricetes*.

Note that the phage LP-LmcIH1-9 of *L. monocytogenes* isolated from the brain membranes was somewhat different from the phage LP-LmcIH1-8 of *L. monocytogenes* isolated from the blood. The deletion was revealed in the gene for the major capsid protein and in the third module, which led to the loss of the ORF of one of the phage proteins.

#### 3.2.4. The CRISPR System

All *L. monocytogenes* ST37 isolates had one CRISPR locus with level of the evidence 4. The locus contained repeating sequences of length of 29 bp with nine spacers. No *cas* genes were found to be associated with this locus, which was located downstream of the ORF of ribose-phosphate pyrophosphokinase in the intergenic region of 1244 bp.

#### 3.2.5. Virulence Factors

A total of 54 virulence factor genes were found in the genomes of *L. monocytogenes* ST37; most of them had the same alleles. In 10 genes, individual isolates had SNV (single nucleotide variant) of the common allele sequences (Table S2). Two isolates (clinical and meat) had no differences in these loci. Two isolates had disrupted the gene *comK* mentioned above. The remaining meat and fish isolates and one clinical isolate had one different locus. Three clinical samples and two isolates from the restaurant cooking environment had two loci with SNV. An increase in the sample size of the ST37 isolates confirmed the previously identified absence of the *vip* gene in *Listeria* of this genotype [18].

#### 3.2.6. Antibiotic Resistance

All ST37 isolates had antibiotic resistance genes only on chromosomes. These genes, *fosX* (lmo1702), lmo0919 (*lin*), lmo1695 (*mprF*), and *norB* (lmo2818), encode resistance to fosfomycin, lincosamides, against cationic antimicrobial peptides, and quinolones. All but one isolate had the same allele of *fosX* (4); the fish isolate GIMC2053:Lmc6646 had an allele with an SNV of allele 4. The meat isolate GIMC2054:Lmc6888 had another allele of the *norB* (31), which was SNV of the common allele 8. All ST37 isolates acquired additional resistance to quinolones via mutations Ser83 and Glu87 in ParC (the subunit of topoisomerase IV). 

Antibiograms provided by clinical laboratories showed resistance of clinical isolates of ST37 to ampicillin and meropenem. So, we analyzed the genes of the penicillin-binding protein (PBP) and beta-lactamases in the genomes of the isolates. All of them had two genes for PBP (1A and 3, COG0768 and COG0744), two genes for beta-lactamase (class C, COG1680), the gene for metallo-beta-lactamase (COG2333), and the gene for metal-dependent hydrolase with the domain of the beta-lactamase (COG1234). On the one hand, PBP3 is the primary target for beta-lactams, and the blocking of these enzymes is lethal for the bacterial cell [38]. However, the affinity of beta-lactams to the other PBPs is variable and does not immediately have a deleterious effect [38]. On the other hand, beta-lactamases could hydrolase beta-lactams, which explains the phenotypic resistance.

## 4. Discussion

In a COVID-19 pandemic, from March 2020 to February 2023, the number of food isolates of *L. monocytogenes* ST37 markedly increased, and cases of invasive listeriosis with manifestations of meningitis and septicemia caused by *L. monocytogenes* ST37 have appeared in the Moscow region. Only male patients 48–81 years old were infected with *L. monocytogenes* ST37. Four of them died, and two survived. In Europe, apart from the sporadic clinical cases mentioned above [4,5,6], one of three large outbreaks in Denmark in 2022 was caused by ST37 (FUD2080) [39]. An additional outbreak of *L. monocytogenes* ST37 was reported, with 8–14 cases accumulating over the last 3–5 years [39]. The sources of these outbreaks remain unknown. Note that other outbreaks in Denmark in 2022 were also caused by *L. monocytogenes* of the PLII, and the number of listeriosis cases per year noticeably increased [39]. The European Centre for Disease Prevention and Control (ECDC) also reported that the number of foodborne outbreaks in 2021 was the highest since the European Food Safety Authority (EFSA) first started collecting data [40]. Two of the four multi-country listeriosis outbreak clusters were caused by *L. monocytogenes* of the PLII (ST7 and ST451) [40].

Analyzing the distribution of invasive listeriosis with manifestations of meningitis and septicemia by gender, we saw that before the COVID-19 pandemic, the male-to-female ratio was 1.2:1 in the Moscow region, but during pandemic, this ratio reached 4:1. In the ECDC 2021 report, male-to-female ratio for confirmed listeriosis cases with known sex in all age categories was 1.2:1. However, in the categories 45–64 years and 65+, this ratio was 1.6:1 and 1.8:1, respectively [39]. Thus, men over the age of 45 are more susceptible to listeriosis than women.

In the Moscow region, *L. monocytogenes* ST37 was isolated from meat and fish products. In European countries, *L. monocytogenes* ST37 was reported in meat and dairy products [11,12,13,14,15,16]. In Austria in 2017, ST37 was the fifth of the ten most frequent STs among the category of food, which included meat, dairy products, and vegetables [41]. The fish products were mentioned as the source of *L. monocytogenes* ST37 in Poland (RTE smoked mackerel paste, 2017). Interesting that core genome of this isolate was identical to the genome of the clinical isolate of 2013, so a possible source of infection was proposed four years later [42].

Pan-genomic studies of the ST37 isolates showed that a different repertoire of accessory, unique, and exclusively absent genes did not affect *Listeria*’s ability to cause disease. The number of unique genes correlated with the number of prophages. Dorscht et al. have suggested that prophages can improve the competitive fitness of the lysogenized host strains [34], but experimentally, the role of prophages was shown only for those that are integrated into the regions of transcription regulators: lmo0112 and *comK*. Hain et al. demonstrated an upregulation of the expression of phage-derived genes when *Listeria* proliferates intracellularly [43], and deletions in the monocin locus of prophages led to severe virulence attenuation [43]. Rabinovich et al. studied the role of the *comK*-integrated prophage in the regulation of phagolysosome escape by *Listeria* during infection. Prophage excision during intracellular growth within phagosomes led to the formation of a functional *comK* gene, and the production of progeny virions was prevented [44].

We described the pseudolysogeny for the strains GIMC2112:LmcIH1-8 and GIMC2113:LmcIH1-9. The locus of integration of the excised prophage is unknown; however, the data of Rabinovich et al. give reason to believe that the excision of the prophage occurred in the stationary phase of growth of *Listeria* cells [44].

The role of the prophages in bacterial adaptation is important when *Listeria* proliferates intracellularly, but this stage is preceded by invasion into enterocytes. The interaction between *Listeria* and enterocytes is prevented by the mucus if it renews and is enriched with health-related bacteria. When the mucus as the first line of defense is damaged, for example, after a viral infection, invasion of enterocytes becomes possible. Therefore, even hypovirulent *Listeria* of the PLII becomes dangerous for people who have had COVID-19 or Influenza.

## 5. Conclusions

The increase in the population’s susceptibility to hypovirulent *L. monocytogenes* raises the importance of molecular-epidemiological studies of *Listeria* of the PLII for the timely identification of the acquired virulence and resistance factors in actively evolving bacteria.

## Figures and Tables

**Table 1:** COG distribution in Pan-genome *L. monocytogenes* ST37.

Groups of the COGs	Genes		
	Unique	Accessory	Core
Cellular processes and signaling	28%	22%	17%
Information storage and processing	30%	32%	14%
Metabolism	14%	14%	40%
Poorly characterized	28%	32%	22%

**Table 2:** Prophages and phages in *L. monocytogenes* ST37 genomes.

Source	Strain	Prophage								Bacteriophage
		lmo0112	tRNA-Ser(cga)	tRNA-Arg(tct)	tRNA-Leu(gag)	comK	tRNA-Arg(ccg)	tRNA-Thr(ggt)	Total	Class: *Caudoviricetes*
FPE	GIMC2026:LmcBD14	√		√			√		3	
FPE	GIMC2027:LmcBD15	√		√			√		3	
Meat	GIMC2054:Lmc6888	√	√	√					3	
Meat	GIMC2071:Lmc9864	√		√			√		3	
Meat	GIMC2106:Lmc10704	√	√	√	√				4	
Meat	GIMC2107:Lmc11363	√	√	√		√			4	
Fish	GIMC2053:Lmc6646	√		√				√	3	
Fish	GIMC2103:Lmc6942	√		√				√	3	
Fish	GIMC2105:Lmc10258	√		√				√	3	
Clin	GIMC2063:LmcUH21	√				√			2	
Clin	GIMC2082:LmcIH1-5	√		√			√		3	
Clin	GIMC2094:LmcH67_7	√		√				√	3	
Clin	GIMC2123:LmcUH37	√		√					2	
Clin	GIMC2134:LmcEH-7	√	√	√					3	
Clin	GIMC2112:LmcIH1-8	√		√				√	3	√
Clin	GIMC2113:LmcIH1-9	√		√				√	3	√

## Data Availability

The data supporting the reported results can be found in BIGSdb-Lm (https://bigsdb.pasteur.fr/listeria/), ID: 76308; 78656; 82484; 98284; 98285; 98295; 102091 (clinical isolates); 42997; 42998; 49371; 49372; 76388; 82493; 98277; 98278; and 98280 (foodborne isolates), and in GenBank NCBI (https://www.ncbi.nlm.nih.gov/genome/, accessed on 14–19 July 2023), Bio Project PRJNA605697, accession numbers: CP127186–CP127191, CP127193, CP127240, CP127241, CP127377, and CP129425–CP129430.

## Supplementary Materials

The following supporting information can be downloaded at https://www.mdpi.com/article/10.3390/life13112167/s1, Table S1: The differences in the core genomes of ST37 isolates; Table S2: Virulence factors that differ among ST37 isolates; and Table S3: Alleles of resistance genes.
